# Editorial Notes

**Published:** 1923-10

**Authors:** 


### Editorial Notes.

**The Centenary Celebrations of the "Lancet."**

THE centenary celebrations of the *Lancet* now in progress mark something greater even than the hundredth birthday of the *Lancet*. It is the centenary not only of one of the greatest of medical journals, but also of medical journalism. The whole history of the *Lancet* is a wonderful example of the possibilities of honest and vigorous individual effort. When Thomas Wakley founded his paper, his main purpose was to bring into the light of publicity certain hole-and-corner practices in the profession which he had but lately joined. His resources consisted of a modest general practice in the neighbourhood of the Strand ; a clear insight into the selfish and dishonest policy of a privileged section of the medical profession of his day ; and an unrivalled capacity for saying what he thought, stimulated by a painful personal experience of injustice and untrammelled by fear of consequences. On this slender foundation he builded better than he knew. The history of his struggles, and of the succession to his policy of those who have followed him, is set forth in the centenary number of the *Lancet,* which every medical man should possess, not only for present reading, but also for future reference. Its policy is now, as it always was, the promotion of honesty in the teaching and practice of medicine. Unsurpassed among medical journals, it remains an example of Editorial acumen and of medical matters written in pure and intelligible English, while it is the model on which many of the most successful medical weeklies have been fashioned. Here it is enough that we offer our congratulations to those now in charge of our illustrious contemporary, who maintain so worthily those traditions of honesty and veracity on which Wakley founded his venture ; for it is a fact, that in medical journalism the *Lancet* maintains its leading position. Those who have been privileged to work for the *Lancet* will bear witness to the consideration always received from those in charge. Our congratulations are therefore no empty form ; they carry with them an acknowledgment of the immense debt which the medical profession in general and that of the English speaking races in particular owes to the *Lancet,* and a belief that the tradition of fearless independence, one of the chief sources of its unequalled influence, will be maintained in the future.

**Service at the Cathedral.**

At the invitation of the Dean of Bristol (the Very Rev. E. A. Burroughs, D.D.), a service was held in the Cathedral on October 21st, the Sunday after St. Luke's Day, at 3.30 p.m.

In spite of the exceptional rain that fell during the afternoon, a large number of medical men resident in Bristol and the neighbourhood attended the service. Many representatives of the nursing profession from the various hospitals in Bristol, private nursing homes, the District Nursing Association and private nurses were also present. The officiating clergy were the Dean, the Sub-Dean (Rev. Canon Alford), the Precentor (Rev. J. M. D. Stancomb), and the Rev. H. A. Watts.

The first lesson (Ecclesiastes xviii. 1–14) was read by Mr. F. Richardson Cross, and the second lesson (St. John xvi.) by the Rev. Canon Alford. The anthem was " Ho, every one that thirsteth, come ye to the waters and buy," from Mendelssohn's *Elijah.*

The sermon was preached by the Dean, who took as his text Hebrews iv. 13 : "Neither is there any creature that is not manifest in his sight : but all things are naked and opened unto the eyes of him with whom we have to do."

The general appreciation expressed of this special service for the medical profession has been such that we understand that the Dean has promised to reserve the Sunday nearest to St. Luke's day for a similar service next year. We trust that so excellent an observance may long continue.

---

